# Functional Roles of 10 Hz Alpha-Band Power Modulating Engagement and Disengagement of Cortical Networks in a Complex Visual Motion Task

**DOI:** 10.1371/journal.pone.0107715

**Published:** 2014-10-06

**Authors:** Kunjan D. Rana, Lucia M. Vaina

**Affiliations:** 1 Boston University, Brain and Vision Research Laboratory, Department of Biomedical Engineering, Boston, Massachusetts, United States of America; 2 Athinoula A. Martinos Center for Biomedical Imaging, Massachusetts General Hospital, Charlestown, Massachusetts, United States of America; 3 Harvard Medical School, Massachusetts General Hospital, Boston, Massachusetts, United States of America; University of British Columbia, Canada

## Abstract

Alpha band power, particularly at the 10 Hz frequency, is significantly involved in sensory inhibition, attention modulation, and working memory. However, the interactions between cortical areas and their relationship to the different functional roles of the alpha band oscillations are still poorly understood. Here we examined alpha band power and the cortico-cortical interregional phase synchrony in a psychophysical task involving the detection of an object moving in depth by an observer in forward self-motion. Wavelet filtering at the 10 Hz frequency revealed differences in the profile of cortical activation in the visual processing regions (occipital and parietal lobes) and in the frontoparietal regions. The alpha rhythm driving the visual processing areas was found to be asynchronous with the frontoparietal regions. These findings suggest a decoupling of the 10 Hz frequency into separate functional roles: sensory inhibition in the visual processing regions and spatial attention in the frontoparietal regions.

## Introduction

Cortical fluctuations between 8–14 Hz (alpha frequency band) are perhaps the most studied brain oscillations since the early days of electrophysiological recordings [1], yet their physiological role remains unclear. Numerous studies involving both direct neuronal recordings and noninvasive EEG (electroencephalography) and MEG (magnetoencephalography) approaches have investigated the nature of this neural oscillation and its contributions to cognitive functions, including memory formation, attention control, and anticipatory recruitment of neurons involved in perceptual or cognitive tasks (for reviews see [2]–[5]). However, the cortical interactions at the 10 Hz frequency and the meaning of such interactions remain poorly understood.

In visual processing regions, the alpha band activity has been identified as a “default state” of the cortex, producing oscillatory power at 10 Hz, which actively inhibits task irrelevant information [6]. For example, in the visual system, there is a reported decrease in occipital alpha-activity during visual attention, and a decrease of visual processing in the context of high alpha activity in the pretask period [7]–[9]. Studies of the primary sensory-motor cortices have suggested that alpha-band, 10 Hz oscillations decrease with attention and movement [10]–[12]. There is also evidence that inhibitory alpha power precedes the involvement of cortical regions in task-related functions [13], [14].

Alpha band activation in the frontoparietal regions has been associated with higher-level processing. For instance EEG studies have shown that alpha power has a spatial bias in the frontoparietal regions [15]–[19] where attending to a visual hemifield will induce contralateral inhibition of alpha power. Contralateral inhibition of alpha power in a region has been linked to functional excitation of that region [4], [20]. This, however, is not a general rule. There is substantial evidence for an increase in alpha power in internally-focused cognitive tasks, such as working and long term memory, different executive functions [16], [21], [22], mental imagery, and mental calculations [5], [23]. While the aforementioned roles have been attributed to the frontoparietal alpha power, it is unclear how this relates functionally to the alpha power observed in the visual processing regions.

In the present MEG study we explored the relationship between alpha power in visual processing regions and in the frontoparietal regions while observers perform a high-level motion task involving the detection of an object moving in depth relative to the scene during an observer's self-motion in depth in a simulated 3D environment. We also investigated the functional role of alpha oscillatory power involved in this task. Similar to the computations described in our previous psychophysical and functional imaging studies, in this task, referred to as Visual Search of a Moving Object by a Moving Observer (VS), optic flow, the global patterns of retinal motion characteristic of the observer's self movement, is identified and subtracted from the retinal flow pattern to isolate the motion of the target within the scene [24], [25]. This process, called *flow parsing*, has been shown to involve global motion processing, not just local motion contrasts [25]–[30]. In our previous fMRI study [25] we computed partial correlations among the regions of interests (ROI) activated when subjects performed the VS task and found four clusters of highly interconnected ROIs. The ROIs in three clusters consisted of typical areas involved in visual or visual motion processing, spanning early visually responsive cortical regions (V1 & V2), regions involved in stimulus motion processing including optic flow(MT+, KO, LO, & V3a), and higher level visually responsive parietal regions presumably involved in the representation of the stimulus (Visual Intraparietal Sulcus (VIP), Dorsal Intraparietal Sulcus middle (DIPSM), and the precuneus). The fourth cluster included frontoparietal regions (Postcentral Sulcus, Postcentral Gyrus, Central Sulcus, and Frontal Eye Field (FEF)). The coarse temporal resolution of fMRI did not allow a more detailed investigation into the relationship among these clusters of activation. Therefore, in this study, using MEG whose high temporal resolution (milliseconds) is excellently suited for exploring the fine spatiotemporal relationship among communicating cortical areas, we investigated the interaction between visual processing regions and the frontoparietal regions. In particular, since there is evidence that alpha-band power plays different roles in the visual processing regions and in the frontoparietal regions, we were interested to determine the functional roles of alpha-band power and when the power increases or decreases during the VS task. We found significant inhibition of 10 Hz alpha power in the visual processing regions after 300 ms relative to the onset of stimulus motion, while in the frontoparietal regions there was a longer, sustained, alpha power, sensitive to target spatial location during the stimulus motion period.

Furthermore, through computing phase synchrony between activated regions of interest, we showed that alpha-band power in the visual processing and the frontoparietal regions are not linked suggesting independence of alpha-band power between these two clusters of cortical areas

## Methods

### 1. Psychophysics

#### 1.1. Participants

Eight healthy volunteers, college students (5 males, age range 18–23 years, mean = 20.125, SD = 1.96) with normal or corrected to normal vision, participated in the study. All participants were right handed according to the Edinburgh Inventory of handedness [31] and none had a history of neurological or psychiatric disorder or medical treatments which might interfere with motor or cognitive performance. Before the experiment, all participants provided written informed consent in accordance with the Declaration of Helsinki (2008) and the requirements of local Ethics Committees on Human Research at Atinoulas Martinos Center, Massachusetts General Hospital and at Boston University which approved this research (IRB Protocol No: 1999P010946 (MGH) and IRB Protocol No: 2387E (BU)). Each subject performed all tasks during a single MEG scanning session. The structural MRI was obtained during a separate scan session.

#### 1.2. Stimuli and Task

Participants performed two tests, the MT+ localization task and a psychophysical task, referred to as Visual Search of a Moving Object by a Moving Observer (VS) involving the detection of an object moving in depth by an observer in simulated forward motion. In both MT+ localization and VS tasks, participants were instructed to maintain fixation on a red circle (0.5 degrees in diameter) placed at the center of the 22″ video display with a resolution of 1650×1080 pixels. Motion in depth was inferred only through changes in object sizes and their motion direction. The display was viewed binocularly but no stereo cues were present. No feedback was provided during the experimental MEG session. Observers entered their response by pressing preselected buttons on an MEG compatible fiber-optic response pad with the fingers of the right hand.

Prior to the MEG scanning session, all subjects were trained on the psychophysical task in the laboratory (at Boston University) and they practiced the task until their performance was significantly above chance (p<0.01).

Visual Search of a Moving Object by a Moving Observer (VS) Task: Stimuli consisted of 9 objects distributed within a 25×25×60 cm volume, centered at a distance of 80 cm. The stimuli were rendered using OpenGL. The objects were high contrast (28.3 cd/m^2^ on a 0.3 cd/m^2^ background) textured spheres with a mean initial diameter of 1.5°. The display area was divided into 9 equally sized wedges, each containing one sphere at a random eccentricity up to 9° (using a square root distribution to create a uniform density), to prevent occlusion between spheres.

**Figure 1:**
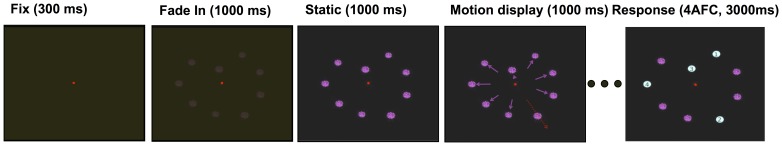
Time course of the VS stimulus. First, the fixation mark appeared on a blank screen and lasted 300 ms. Next, the 9 textured spheres faded in from the background over a 1000 ms period. Then, the spheres remained static for 1000 ms. This was followed by displaying the stimulus motion for 1000 ms, 8 spheres simulating forward observer motion and the other (target) had independent motion, forward or backward with different speeds than that o the observer. Finally, the response period, lasting 3000 ms, displayed the spheres static, four of which were labeled with numbers 1–4, one corresponding to the target. The subject's task was to chose the number corresponding to target.

The time flow of the experiment was as follows (Figure 1). Each trial began with a 300 ms blank screen. Over the following 1000 ms the contrast was ramped up from 0% so that, at the end, the 9 spheres were clearly visible. During the next 1000 ms the spheres remained visible and stationary. In the next 1000 ms (the stimulus-motion period), the spheres were moved and scaled consistent with forward observer translation of 3 cm/sec (relative to a 30 cm simulated distance to the spheres, such that the radial velocity was up to 1.66°/sec for the most eccentric objects, or 0.84°/sec for spheres of mean eccentricity). The beginning of the stimulus-motion period defined the 0 ms marker for each trial. One of the spheres (the “target”) had an independent forward or backward motion vector of 2, 4, 6 or 8 cm/sec within the scene in addition to the induced self-motion described above. Throughout the motion, subjects had to monitor all 9 spheres since the labels did not appear until after the end of stimulus motion. After the 1000 ms motion display, the spheres were displayed static for 3000 ms and the target and three other randomly selected spheres were labeled with numerals, 1–4. In a four-alternative forced choice (4AFC) task, observers identified the target sphere by pressing on the response pad one of the four buttons, 1–4, corresponding to the location of the target sphere. After 3000 ms the static spheres disappeared from the screen and a new trial started. The timeline of these events is illustrated in Figure 1. Subjects were presented 2 consecutive runs of the VS task, 80 trials each (160 trials total), evenly interleaving each target speed (20 trials per speed) in a random order in each run.

MT+ Localization (MTLoc): A blank screen was shown for 300 ms, followed by nine textured spheres (1.5 degrees in diameter) which faded into the screen for 1000 ms. Next, for another 1000 ms, the spheres were displayed static followed by 1000 ms radial motion, (expansion or contraction), simulating an observer walking forward or backward on a straight trajectory (with a speed of 3 cm/s). In a two-alternative forced choice (2AFC) task, observers reported the direction of the stimulus-motion by pressing predefined keys on the response pad. There were two consecutive runs, 80 trials each (160 trials total), with forward and backward motion trials evenly interleaved (across the two runs, 80 trials expansion and 80 trials contraction).

The VS task involves both self-motion of the observer and the motion of the target. To compare the target detection in the VS task to self-motion only, we used as a control the expanding motion trials of the MT+ Localizer task (Exp Only). The Exp Only trials were visually equivalent to those in the VS stimulus except there was no independently moving sphere (the target) and also after the end of the stimulus-motion period, when the spheres were static, no labels (numbers 1–4) were displayed. Exp Only portrayed only the forward motion of the observer and thus the response was to the direction of motion, while in the VS task the response was to the location of the independently moving sphere (the target).

#### 1.3. Behavioral data analysis

Response and reaction time were recorded for each trial in every subject. In the VS task, reaction times were measured as the time difference between the appearance of the numbered labels to when the subject made the button press to indicate his/her choice of the target (1000–4000 ms after stimulus motion onset). A trial was treated as having no response, and discarded, if the subject did not respond within the 3000 ms allocated for response. All subjects performed significantly above chance (25%) (p<0.01). Most of the correct responses occurred within the first 1000 ms of the response period, discussed in section 1 of the Results (Behavioral Results).

### 2. MEG Processing Methods

In this section we outline the steps for acquiring and processing the MEG data. Each MEG run was divided into epochs defining the time evolution of each trial. The MEG data were registered to an anatomical MRI volume to map MEG sensor data onto the cortical surface of each subject's brain. Regions of Interest (ROIs) were extracted through finding clusters of activation in the mapped activation and then pruned by using a resolution matrix to eliminate from the analysis regions of high cross-talk (discussed in Section 2.5 of Methods).

#### 2.1. MEG data Acquisition

The MEG study was conducted at the Athinoula A. Martinos Center for Biomedical Imaging in Charlestown, Massachusetts. Participants were seated in upright position under the MEG dewar and faced the projection screen placed 80 cm away measured from the eyes. The MEG data were acquired with a 306-channel Neuromag Vectorview whole-head system (Elekta Neuromag Finland) comprising 204 orthogonally oriented planar gradiometers and 102 magnetometers at 102 locations. The system was housed in a three-layer magnetically shielded and sound-proof room (Imedco AG, Switzerland). During data acquisition the room was darkened.

We used an LP350 DLP projector (InFocus, Wilsonville, OR) at a resolution of 1024×768 pixels with refresh rate of 75 Hz in the MEG scanning room to present the stimulus. To compute the head position inside the MEG, four head-position indicator (HPI) electrodes were affixed to the subject's head. The positions of the HPI electrodes on the head and at least 80 points sampled on the scalp were entered with a magnetic digitizer (Polhemus FastTrack 3D) in a head coordinate frame defined by anatomical landmarks, which included the nasion and the left and right auricular points. Vertical and horizontal electro-oculogram (EOG) measurements were also recorded to monitor eye-movements and blinks. Trials contaminated by artifacts, such as eye blinks, sensor jumps, or loss-of fixation were rejected. The MEG signals were band-pass filtered to the frequency range 0.5–200 Hz and digitized at 600 samples/s (600 Hz).

#### 2.2. MEG epochs

For each trial the MEG signal was divided into epochs with the 0 ms mark placed at the onset of the motion stimulus. Each epoch extended from −500 to 2000 ms relative to the onset of the motion stimulus. This time interval consisted of the prestimulus period when the spheres were stationary (see VS task description in section 1.2 of Methods) (−500 ms to 0 ms), the stimulus-motion period (0 to 1000 ms), and the first 1000 s of the response period (1000–2000 ms). We truncated the 3000 ms response period because most responses (button box press) occurred in the first 1000 ms (discussed in Section 1 of the Results).

Epochs were rejected and removed from the dataset if corresponding gradiometer readings exceeded 2 pT/m peak-to-peak or magnetometer readings exceeded 6 pT peak-to-peak. Such large signal fluctuations are due to head or eye motion artifacts and cannot be easily filtered out from the data. In this study, for each of the participating subjects, the maximum number of trials rejected was less than or equal to 3 (below 2% of the total number of trials) leaving between 157–160 total epochs per condition per subject. Eye blink artifacts were removed by first computing an SSP operator from −200 ms to 200 ms of the DC-removed, raw signal centered at 20 to 30 peaks of vertical EOG channel recordings and then by removing the first principal component of the gradiometer and magnetometer readings. We normalized the signal by subtracting the mean and dividing by the standard deviation of the signal in the prestimulus.

#### 2.3. Anatomical MRI and MEG spatial registration

T1 weighted structural MRI scans were acquired on a separate day using an 8-channel phase array head coil in a 3T scanner (Siemens-Trio, Erlagen, Germany). Parameters of the sequence were: distance factor: 50%; slices per slab: 128; FOV: 256; FOV phase: 100; slice thickness: 1.33 mm, TR: 2530 ms, TE: 3.39 ms. Freesurfer software (http://surfer.nmr.mgh.harvard.edu) [32]–[35] was used for cortical reconstruction and volumetric segmentation of the T1 weighed whole brain images for each subject. The individual brain scans were motion corrected, spatially co-registered by morphing into the Freesurfer average brain through spherical surface mapping [36] and spatially smoothed with a 5 mm FWHM (Full with at Half Maximum) kernel.

To perform the alignment of each individual subject MEG data onto their corresponding structural MRI of the brain, we used the MNE software (http://www.nmr.mgh.harvard.edu/martinos/userInfo/data/sofMNE.php) [37]. We matched the fiduciary landmarks to their respective locations within the reconstructed skin surface from the anatomical MRI scan. The alignment was refined by applying the iterative closest point algorithm [38].

#### 2.4. MEG source estimates

Spatial distributions of cortical currents underlying the MEG signals were estimated using the L2 minimum-norm approach computed with the MNE software. The sources were restricted to the grey matter surface extracted with the FreeSurfer software. The triangulation of the cortex was decimated to about 9000 sources per hemisphere resulting in an average distance of 4 mm between adjacent sources. The orientations of the sources were approximately constrained to the cortical surface normal direction using the loose orientation constraint approach. The noise covariance matrix was estimated from the prestimulus period (−500–0 ms). The dynamic Statistical Parametric Map (dSPM), computed for each subject, was obtained by normalizing source estimates by the noise covariance matrix. We used the dSPM to select clusters of activation (as described in Section 2.5 of Methods).

#### 2.5. Regions of Interests (ROIs)

The MT+ Localization test was used to localize the motion-sensitive MT+ area. We isolated the ERF equivalent to the P230 corresponding to radial motion [39]. Since our motion was complex radial motion, we expected MT+ to be activated at this signal peak. Time courses were bandpass filtered from 0.5 to 40 Hz to reduce high frequency noise and sensor drift to isolate the P230 peak. The peak within 20 ms of 230 ms relative to motion onset was labeled as the P230. The MT+ region of interest (ROI) was chosen through manual extraction of the activated cluster in middle temporal area at least 2.5 SD above the noise level.

All other regions of interest (ROIs) were generated through manual extraction of activation clusters from the averaged MEG data morphed onto the Freesurfer average brain (fsaverage) [36] in the VS task. Anatomical names of areas are based on the FreeSurfer parcellation and locations of activity within each parcellation region. Principal Components Analysis was performed across the three-dimensional source data points within each ROI to obtain the principal dipole orientation. Activation time courses for each ROI were computed on a trial-by-trial basis through a spatial average of the activation projected along the principal dipole orientation.

Together, the effect of the sensors mapped to thousands of cortical surface locations and the smoothing performed in the MNE source space solution lead to a significant cross-talk between some ROIs time courses. To minimize the amount of cross-talk between ROIs, we isolated those ROIs with significant signal spread, and removed them from the analysis. We evaluated systematically the cross-talk between the activated cortical areas, by constructing a resolution matrix (adapted from [40]) to measure how one ROI's signal maps into another ROI's signal. The point spread function on a measured dipole strength vector 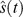 relates to the true strength s(t) as follows:
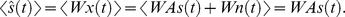



In the expression above, the measured source data were computed from the sensor data x(t) transformed to the source space via the inverse transform operator W. We modeled x(t) as the summation of the true dipole strength s(t) transformed onto the sensor space via the forward transform operator A with an added sensor noise n(t). The matrix WA is the resolution matrix, where columns specify the pointspread for each dipole location.

We performed a similar operation for measuring signal spread between ROIs. We define W_ROI_ as the inverse transform operator mapping from sensors to ROIs and A_ROI_ as the forward transform operator mapping from ROIs to sensors. To compute the columns of W_ROI_, each corresponding to the projection from the sensors onto a particular ROI, we projected the columns of the inverse operator W onto the principal dipole direction of the ROI and averaged the vertex components within the ROI. Similarly, we computed the rows of A_ROI_ by projecting each ROI's principal dipole orientation onto the rows of forward operator A. We computed the resolution matrix R = W_ROI_A_ROI_. The matrix R was normalized by dividing each column of matrix R by the column sum to form R*_rel_*, the normalized resolution matrix where each cell represents the percent signal contribution of the ROI, corresponding to the row, on the signal of the ROI corresponding to the column.

We computed the average resolution matrix across all the subjects:
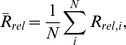
where R_rel,i_ is the normalized matrix R above for subject i and N is the total number of subjects.

We removed from further analysis the ROIs whose signal contributions from any other region was more than 20%. However, when two ROIs had strong cross-talk with each other (>20% contribution), then they were joined into a single ROI. The ROIs whose true signal contributions to the measured ROI signal was less than 20% were removed.

While the procedure described above does not solve entirely the cross-talk problem, it reduces its effect on the ROIs ultimately included in our analysis.

### 3. Data Analysis Methods

In this study we investigated the spatiotemporal profile of the alpha band power at 10 Hz and its role in the functional linking between visual motion processing regions and frontoparietal regions in a complex visual motion task (VS). First we used wavelet decomposition to compute the oscillatory power in time at the 10 Hz alpha frequency (discussed in Section 3.1 of Methods). Second, in order to measure how the 10 Hz oscillatory power is synchronized among ROIs, we computed the Weighted Phase Lag Index (discussed in Section 3.2 of Methods).

#### 3.1. Frequency Bands (Wavelets)

To compute time-varying 10 Hz alpha-band oscillatory power, we applied the complex Morlet wavelet filter [41] to the ROI's time courses using the Matlab (MathWorks, Natick, Ma) package Fieldtrip (http://fieldtrip.fcdonders.nl) [42]. We were interested in determining how the 10 Hz power in the VS and Exp Only conditions varied over time. To compute the raw 10 Hz signal, we first extracted trial-by-trial epochs from −500 to 2000 ms relative to sphere-motion stimulus onset in both conditions. The average prestimulus signal (−500–0 ms) was subtracted from each epoch. Individual epochs were then filtered by the Morlet wavelet filter (10 Hz, 7 cycle bandwidth) to produce the 10 Hz alpha power in each epoch. Wavelet filtered time courses for each ROI were averaged together to obtain the induced 10 Hz alpha-band oscillatory power.

Since the activation in each ROI depends on the size of the region and on the sensor mappings into that ROI, the raw field strength mapped to an ROI was not useful for comparisons between two activated regions. The dSPM solution normalizes the signals across the cortical vertices, but the averaged ROI signal would not be appropriately normalized. We are interested in the strength of prestimulus 10 Hz power, therefore normalization must be computed independently from the prestimulus period. In addition, to be able to compare the 10 Hz alpha power to other frequencies, we need to normalize power across frequencies since measured MEG recordings tend to have an inverse frequency power spectrum [43]. We normalized the signal, sample-by-sample, along frequencies by fitting each ROI's signal power spectrum to a generalized inverse function of the form P = β/(f^γ^) where P is power, f is frequency, and β and γ are functional parameters. The values of β and γ were determined by a linear fit of the log of P over the frequency range of 5 Hz to 60 Hz. This operation is necessary so that the other frequencies can be compared to the 10 Hz signal. After normalizing across frequencies, we standardized the signal sample-by-sample to a pseudo z-score, which allowed comparison of power between ROIs. To compare the signals sample-by-sample to a standard baseline within the alpha band, we further normalized the signals' wavelet power coefficients through the mean and variance of baseline (−500–0 ms) wavelet coefficients at the frequencies 6–14 Hz in 1 Hz steps. We chose only the wavelet coefficients that were free from distortion effects, that is those whose wavelet kernel spanned values in the −500 to 2000 ms range. Due to the 7-cycle kernel size, the wavelet kernel at 10 Hz will span +/−350 ms. Thus, the computed 10 Hz signal begins at −150 ms instead of at −500 ms and ends at 1650 ms instead of at 2000 ms.

#### 3.2. Phase Synchrony

Following up on Palva&Palva's [5] hypothesis that alpha synchronization among frontoparietal areas does not extend to visual processing regions, we set out to determine how the 10 Hz power is functionally linked to the different ROIs active during the VS task compared to the control (Exp Only). We used the Fieldtrip toolbox (http://fieldtrip.fcdonders.nl) [42] to compute synchrony in the time-frequency domain by using the debiased weighted phase lag index (WPLI) [44]. The WPLI method is ideal for computing synchrony between cortical regions in MEG because this measure is invariant to linear mixing effects from ROI cross-talk and, through the weighting of the imaginary component, is more resilient to noise than the traditional Phase Lag Index (PLI) method. Thus, cross-talk will not generate additional false-positives in the WPLI analysis, which, however, may still remain, in small quantity after pruning ROIs with the resolution matrix (described in Section 2.5 of Methods).

The WPLI uses the imaginary components of the cross-spectral density (CSD) between two ROIs. We computed CSD between a pair of ROIs sample-by-sample across time as the average of the trial-by-trial product of the wavelet coefficient of one TFR with the complex conjugate of another ROI's wavelet coefficient for a particular time-frequency point. The trial-by-trial imaginary component 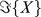 and the phase angle θ of the CSD for each sample was used to compute the WPLI statistic:
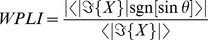



The standard error measure of the mean (SEM) was computed from the mean of the jackknife [45] distribution of the wavelet coefficients. The resulting WPLI measure was divided by the SEM to obtain a pseudo z-score significance statistic [44]. We combined pseudo z-scores across all subjects using Stouffer's method [46]. Then, we determined the time intervals with significant synchrony by applying cluster permutation testing. The statistic of the permutation incorporates both the strength of synchrony and the length of the time window of synchrony. We defined the threshold for determining when time intervals of phase synchrony begin and end as the mean synchrony values across time and across all ROIs. The statistic of the permutation was defined as the integral of the pseudo z-scores across a time interval. We performed 5000 permutations of epoch ordering amongst all ROIs and pooled together the z-score time interval cluster statistics as defined previously. The resulting pooled cluster statistics forms an empirical null distribution. We computed the significance (p-value) of the true, non-permuted cluster statistic as the ratio of permuted statistics that were larger than the non-permuted statistic to the total number of permuted statistics. This procedure provided a significance score that takes into consideration the length of time the pseudo z-score measuring synchrony was sustained above the average pseudo z-score and how significant the score was on average over the time interval.

## Results

### 1. Behavioral results

Behavioral performance was assessed as a percentage of correct responses for object motion (target) detection in the VS task, and for discrimination of the direction of the pattern of radial motion (expansion or contraction) in the MT+ Localizer (MTLoc) task. We also recorded the reaction time in each trial. In the MTLoc task, the detection rate was close to 100% correct in all subjects. In the VS task the mean percent correct of target detection was 50.8%+/−7.6% (2 SE), which is significantly above chance (p<0.01). Most (78.2%) correct responses in the VS task occurred between 1400 ms and 2000 ms after the start of the motion stimulus (400–1000 ms relative to the beginning of the response period). Therefore, for all data analysis, we truncated the MEG time course at the 2000 ms mark (1000 ms stimulus motion period, and 1000 ms of the response period).

### 2. ROI selection

**Figure 2:**
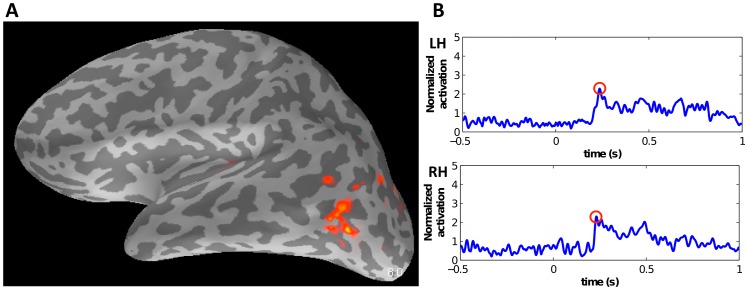
MT+ 230 ms ERF on the cortex. (A) Left hemisphere cortical activation at 230 ms of MT+ area in a representative subject. The activation shown on the inflated brain, in the cortical region, is at least 2.5 SD above noise level. (B) The corresponding time courses of MT+ activation in the same subject (top: left hemisphere; bottom: right hemisphere). The red circle indicates the peak at the 230 ms ERF corresponding to P230.

All ROIs were extracted manually as described in Section 2.5 of the Methods. The MT+ region was identified functionally through the MT+ Localizer (MTLoc) task. We manually drew ROIs for the locations of activation peaks near the expected P230 ERF in MT+ where activation was at least 2.5 SD above the noise level (Figure 2). We found activation in the MT+ within 20 ms of 230 ms, corresponding to the P230, which has been shown to be sensitive to radial motion patterns [39].

**Figure 3:**
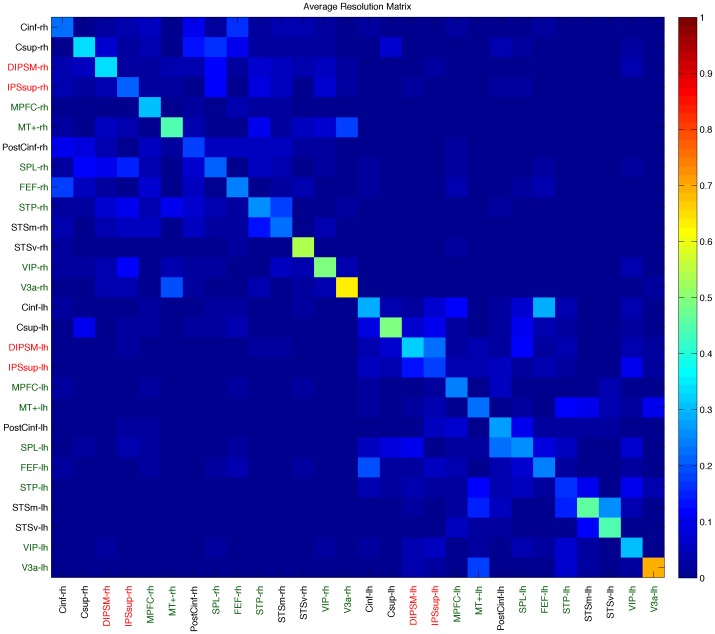
Resolution matrix showing percentage signal contribution across columns of each ROI. ROIs marked in red are joined together, ROIs in black were discarded, and ROIs in green are considered as separable regions. Abbreviations: Cinf – Inferior Central Sulcus, Csup – Superior Central Sulcus, DIPSM – Dorsal intraparietal sulcus middle, IPSsup – superior Intraparietal Sulcus, MPFC – middle Prefrontal Cortex, MT+ - human middle temporal area, PostCinf – Inferior Postcentral sulcus, SPL – Superior Parietal Lobule, FEF – Frontal Eye Field area, STP – Superior Temporal Polysensory area, STSm – Middle Superior Temporal Sulcus, VIP – Ventral Intraparietal Sulcus, V3a – area V3a.

**Figure 4:**
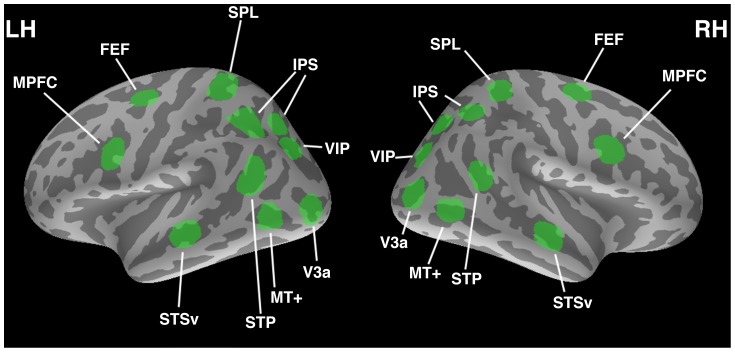
Cortical Areas in the left and right hemispheres that remained after the application of the resolution matrix. Abbreviations: STSv: ventral part of the Superior Temporal Sulcus; STP, the Superior Temporal Polysensory area; MT+: human Middle Temporal determined with the MToc test. It includes areas MT, MST, and V6; V3a: is retinotopic area V3a that was defined through fMRI retinotopic mapping,;VIP: Ventral Intraparietal area,;IPS: Intraparietal Sulcus;, SPL: Superior Temporal Lobule,; FEF:Frontal Eye Field: and MPFC: Middle Prefrontal Cortex.

The other ROIs were defined based on the location of the centroid of measured cortical activation regions on the Freesurfer anatomical parcellation. To minimize cross-talk between ROIs and to find minimally-correlated areas we computed a resolution matrix (Figure 3). Areas with overlap more than 20%, were combined into one ROI (ROIs marked in red in Fig. 3), while areas with significant cross-talk with other ROI's (>20%) were removed (ROI's marked in black in Fig. 3). The PostCinf region in the right hemisphere was also removed due to having less than 20% signal contribution from its own area. Figure 4 illustrates the cortical areas that remained after pruning the set of ROI's with high crosstalk or low power. Since areas IPSsup and DIPSM in the left hemisphere had strong cross-talk, we combined them into one area, IPS (Figure 4). The corresponding areas in the right hemisphere were also joined together to make equivalent regions in both hemispheres. The Cinf, Csup, PostCinf, STSm regions were removed in both hemispheres because they had large signal contributions from neighboring regions.

### 3. Alpha-Band Wavelet Power

**Figure 5:**
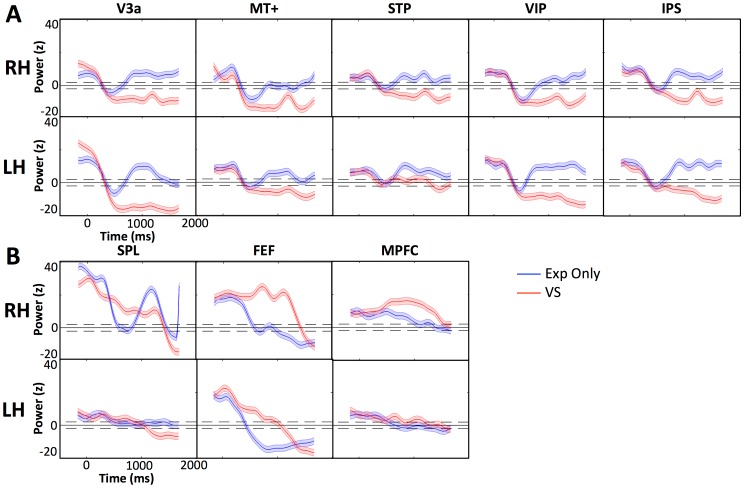
Time courses of 10 Hz alpha band oscillatory power in selected ROIs. ROIs include left (LH) and right (RH) hemispheres in Exp Only and VS (A) visual processing areas and (B) frontoparietal areas. The y-axis represents the z-score of oscillatory power. The x-axis represents time in milliseconds relative to motion onset (0 ms). The blue line shows the 10 Hz power in the Exp Only condition with 2SD error shown in blue shade around the time course. The red line shows the 10 Hz power in the VS condition with 2SD error in red shade around the time course. The black horizontal line indicates z = 0, representing the average normalized baseline power. Dotted lines indicate z = 2 and z = −2 lines.

Specific functions of 10 Hz alpha power have been shown to be spatially localized. For instance, visual processing region alpha power has been linked to inhibition of function. Alpha power in the frontoparietal regions have been linked to modulatory processing of attention and working memory [16], [47]–[50]. Regions where 10 Hz alpha power was significant (z>3), were grouped into specific ROI clusters of similar function (visual processing ROIs and frontoparietal ROIs). To investigate when alpha power was in a deactivated state, we determined when the 10 Hz power dropped to levels consistent with the rest of the alpha band (−3<z<3), which we define as the baseline activation level. Figure 5 shows 10 Hz alpha power in the visual processing ROIs and the frontoparietal ROIs. We recorded the intervals when the VS task and the Exp Only control task signals rose above the baseline (z>3). We also recorded when the signals in VS task and Exp Only control task diverged, which is when the signals became significantly different from each other. This was computed as the time intervals at which the VS signal was 4 SE away in power from the signal in Exp Only. The time periods of 10-Hz alpha power signal rising above the baseline in both Exp Only and VS conditions and the divergence of these time courses are reported in Table 1. Given the inhibitory role of alpha, reaching baseline levels would imply that the region had been released from inhibition. Such time periods in ROIs can be considered deactivated 10 Hz alpha power states. We found two groups of regions with differing activation profiles: one including visual processing ROIs (V3a, MT+, STP, VIP, IPS) and, the other, the frontoparietal ROIs (SPL, FEF, MPFC). The visual processing areas had similarly timed, significant drop-offs of alpha power early in the VS stimulus (around 300 ms) whereas frontoparietal ROIs had longer sustained alpha power lasting through the stimulus-motion period and dropping off in the response period.

**Table 1:** 10 Hz Alpha Power time periods above threshold.

	LEFT HEMISPHERE			RIGHT HEMISPHERE		
Label	Exp Only	VS	Divergence	Exp Only	VS	Divergence
V3a	−150–304 ms	−150–288 ms	−150–131 ms	−150–216 ms	−150–243 ms	−150–11 ms
	735–1323 ms		389–1649 ms	720–1650 ms		462–1649 ms
MT+	−150–324 ms	−150–301 ms	621–1650 ms	−150–301 ms	−150–249 ms	−150–110 ms
	750–1295 ms			1590–1650 ms		19–1650 ms
	1596–1650 ms					
STP	−150–342 ms	−150–259 ms	702–1650 ms	−150–268 ms	−150–294 ms	579–1650 ms
	629–1650 ms			694–1316 ms		
				1465–1650 ms		
VIP	−150–317 ms	−150–299 ms	502–1650 ms	−150–283 ms	−150–281 ms	642–1650 ms
	622–1650 ms			975–1202 ms		
				1296–1650 ms		
IPS	−150–316 ms	−150–296 ms	621–1650 ms	−150–327 ms	−150–308 ms	612–1650 ms
	669–1650 ms			679–1650 ms		
SPL	−150–437 ms	−150–805 ms	1108–1650 ms	−150–521 ms	−150–1361 ms	−150–4 ms
				855–1403 ms		143–372 ms
				1641–1650 ms		427–900 ms
						975–1650 ms
FEF	−150–384 ms	−150–925 ms	−69–241 ms	−150–511 ms	−150–1371 ms	327–1495 ms
			319–1260 ms			
			1445–1650 ms			
MPFC	−150–479 ms	−150–447 ms	599–770 ms	−150–1002 ms	−150–1506 ms	489–1505 ms
		487–762 ms				
		977–1137 ms				

Exp Only and VS columns indicate time periods where the signal is 3 SD above threshold. Divergence columns indicate time periods where Exp Only and VS signals diverged (over 4SD time course). Recordings occurred as early as −150 ms and as late as 1650 ms relative to motion onset.

### 4. Specificity of 10 Hz Alpha Power as a function of object location and task difficulty

**Figure 6:**
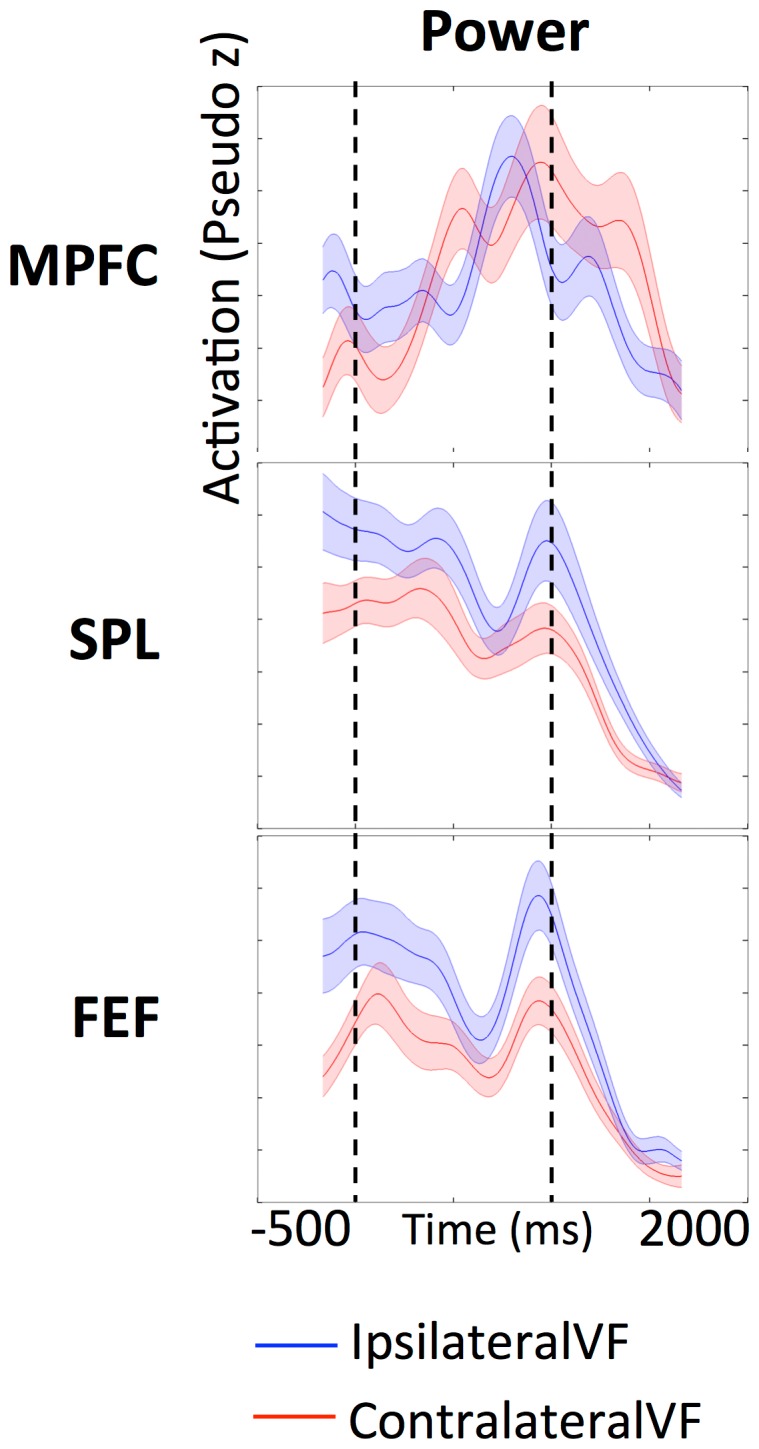
Wavelet power at 10 Hz in frontoparietal regions. The x-axis represents time from stimulus motion onset and the y-axis represents the pseudo z-score alpha power in the region. The blue line illustrates activation in ipsilateral VF and the red line, activation in the contralateral VF. The shaded region indicates 2 standard errors about the mean of each time course. Dotted lines denote 0 ms and 1000 ms relative to motion onset.

To determine the contribution of the frontoparietal network to spatial attention processing we examined how alpha power in the ROIs of this network varied with target location in the VS task. We constrained the wavelet computation by comparing the trial-by-trial averages for when the target sphere was in the observer's visual hemifield opposite to the hemisphere of the cortical areas measured (ContralateralVF) to those where the target was in the same visual hemifield as the hemisphere in which activity in the cortical areas was measured (IpsilateralVF) (Figure 6). Figure 6 shows that both SPL and FEF were sensitive to object location. Throughout the −500 ms to 2000 ms time window, in both IpsilateralVF and ContralateralVF, 10 Hz alpha-band power in SPL and FEF was consistently lower for the target presented in the ContralateralVF (SE>2) (Figure 6). This clear cut difference of activation elicited by the presence of the target in specific spatial locations was absent in MPFC where alpha power overlapped throughout the stimulus-motion period (0–1000 ms), irrespective of whether the target was in the ipsilateral or contralateral visual field.

### 5. Phase Synchrony

We reported the characteristic drop-offs of 10 Hz alpha-band power in visual processing regions and the characteristic sustained activation alpha-band power in the frontoparietal regions (Section 3 of the Results). Here we discuss the patterns of 10 Hz alpha synchrony between ROIs activated in each of the two networks in the VS task. We computed phase-synchrony using WPLI and assessed whether subgroups of ROIs were coactivated with consistent phase across trials. Consistent coactivation implies that either the ROIs intercommunicate or that they are jointly driven by a third neural oscillator [44], [51].

**Figure 7:**
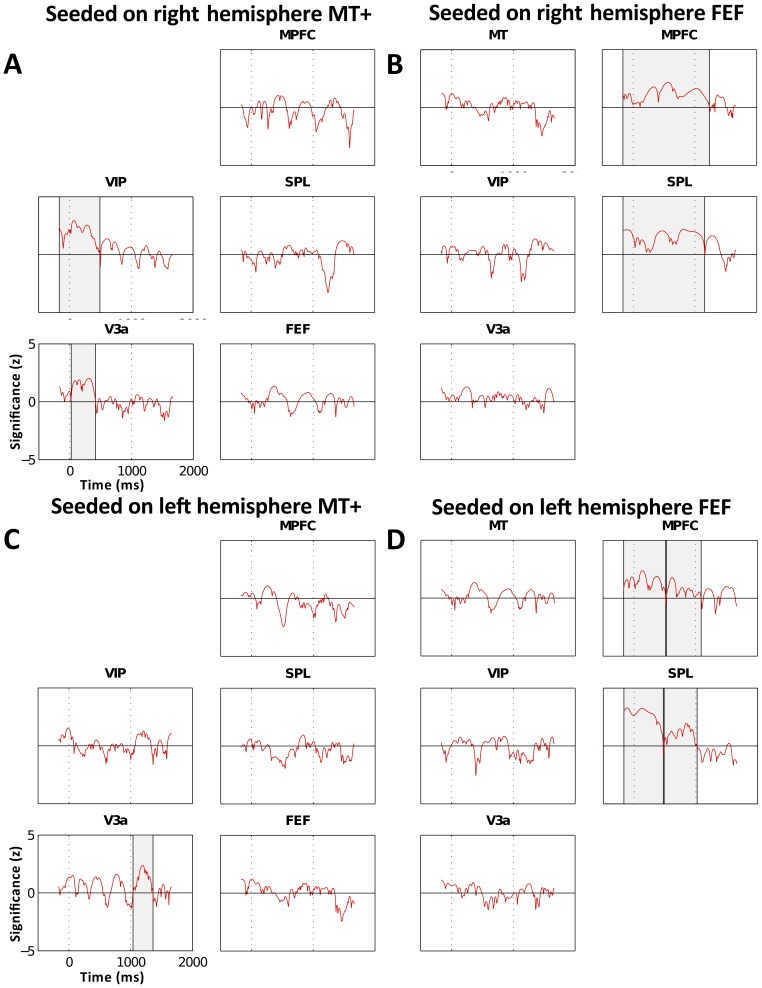
Weighted Phase Lag Index in select ROIs. ROIs are seeded on (A) right hemisphere MT+, (B) left hemisphere MT+, (C) right hemisphere FEF, (D) left hemisphere FEF in the VS task normalized by the within-frequency standard error measure (SEM). The x-axis represents time and the y-axis represents pseudo z-score significance. Shaded regions indicate regions of p<0.01 cluster significance.

To determine when and what regions were phase-synchronized in the VS task, we computed between each pair of ROIs the WPLI score across time at the 10 Hz frequency. Figure 7 illustrates the phase synchrony in VS at 10 Hz between the active ROIs seeding on MT+ and on FEF. Human neuroimaging studies and nonhuman electrophysiological studies reported that MT+ is a critical area for processing motion information [52], [53] and that FEF is a key area for planning saccades towards a visual search target and for covert shifts of attention [47], [54]. Each ROI's time course represents the time-varying pseudo z-score significance of synchrony with the seeded region. Cluster significance plotted in Figure 7 as gray shaded regions show that the visual processing areas MT+-VIP-V3a tended to be synchronized early in the stimulus-motion period (<500 ms) while MPFC-SPL-FEF were synchronized throughout the stimulus-motion period (0–1000 ms). This pattern of activation was observed in both hemispheres, but was higher in the right hemisphere. There was also additional visual processing synchronization in the left hemisphere during the response period (1000–2000 ms).

Figure 7A illustrates the time intervals of connectivity between MT+ and the other visual processing ROI's. In the right hemisphere MT+ and VIP were significantly synchronized between −150 ms to 494 ms (p<0.001) and MT+ and V3a between 25 ms–415 ms (p<0.001). Areas V3a and VIP were synchronized between −150 ms to 694 ms (p<0.001). This pattern of synchronization was weaker and shorter lasting in the left hemisphere (Figure 7C), where there was early synchronization starting at −150 ms and ending at 80 ms between MT+ and VIP (p = 0.039) and at 108 ms between MT+ and V3a (p = 0.038). Since the clusters were above the p = 0.01 threshold, they were not shaded in Figure 7, however, they follow the same early synchrony pattern as the right hemisphere VIP and V3a ROIs. The visual processing regions connectivity (V3a, VIP, and MT+) present in the left and right hemispheres was consistent with the early high activation in the alpha-band which dropped off early in the stimulus-motion period (around 300 ms).

Figure 7B shows significant and long lasting synchrony between FEF and MPFC in the right hemisphere between −150 ms to 1235 ms (p<0.001). Similar synchrony was also seen between FEF and SPL (−150–1158 ms, p<0.001) and between SPL and MPFC in two, almost contiguous time intervals (327–795 ms, p = 0.004, 820–1431 ms, p = 0.002). In the left hemisphere, synchrony clusters were separated by a small but very short zero crossing with synchrony between FEF and MPFC in the −150–1088 ms interval (−150–516 ms, p<0.001, 528–1088 ms, p<0.005) and between FEF and SPL in the −150–1020 ms interval (−150–472 ms, p<0.001, 487–1020 ms, p = 0.002). However, synchrony between MPFC and SPL was significant only in the time interval 91–565 ms (p = 0.006). In general, the frontoparietal regions were synchronized during all or most of the stimulus-motion period (0–1000 ms). This is different from the visual processing regions that were mostly synchronized in the early part of the motion stimulus (<500 ms).

## Discussion

In this study, we investigated the dynamics of 10 Hz alpha-band power and synchrony in an experimental paradigm involving search in a complex motion task (VS) which we studied previously psychophysically [24] and in fMRI [25]. We showed the separability of 10 Hz alpha-band power synchronization between visual processing regions and the frontoparietal regions as well as differing properties of the activation profiles within the regions between the VS and control (Exp Only) tasks. In this section we discuss how these functions relate to behavioral performance and further interpret the functional role of the 10 Hz alpha power in the VS task. We first discuss the inhibitory role of the 10 Hz alpha power in the visual processing ROIs in Section 1 below. Then, we discuss the separability of the 10 Hz alpha synchronization among the visual processing and the frontoparietal ROIs and suggest processing roles for the separate networks in Section 2. Finally, in Section 3, we discuss the frontoparietal alpha power and synchronization and interpret their functions in the context of spatial attention.

### 1. Inhibitory Role of 10 Hz Alpha power in Visual Processing Areas

In the first 300 ms of the stimulus motion, high 10 Hz alpha power was present in both the VS and Exp Only experimental conditions. Starting at approximately 300 ms after stimulus motion onset, there was a decrease in the 10 Hz alpha power (below z = 3 baseline) in the visual processing ROIs in both hemispheres. In the VS task only, this low alpha power remained sustained into the response period (>1000 ms) (Section 3 of Results). In the Exp Only condition, alpha power increased again before the beginning of the response period (<1000 ms). One possible explanation of this difference in the two experimental conditions is that in VS subjects were engaged in detecting a moving object and maintaining in working memory a representation of the location of the candidate targets up to the moment of decision making, that is of target selection. In the Exp Only condition, subjects' task was only to discriminate the direction of the motion of the spheres. The results were congruent with the alpha band inhibition hypothesis [6]. In the VS task, subjects were engaged, and thus 10-Hz alpha power remained low while in the Exp Only condition, 10-Hz alpha power rose quickly back to baseline levels, as there was no demand on the subject's sustained attention and working memory. V3a in both hemispheres and MT+ in the right hemisphere had significantly higher (SE>4 relative to VS signal) alpha power in the prestimulus of the VS task, than of the Exp Only condition. Prestimulus alpha power has been found to be inversely proportional to performance and evoked response in sensory cortices [13], [14]. However, there are studies showing that prestimulus alpha power in the parietal cortex is positively correlated with performance [55] and with a post-stimulus ERP [56]. We suggest that the low prestimulus alpha power in the Exp Only condition was due to the almost exclusive involvement of the visual processing regions whereas the VS task required recruiting the frontoparietal regions as well. However, it is important to note that the alpha power in the right-hemisphere MT+ is only significantly higher in VS during a short time span (−150 ms to −110 ms) whereas the difference in V3a is maintained close to or past the onset of the motion in the stimulus (−150 ms to 131 ms in the left hemisphere and −150 ms to −11 ms in the right hemisphere).

**Figure 8:**
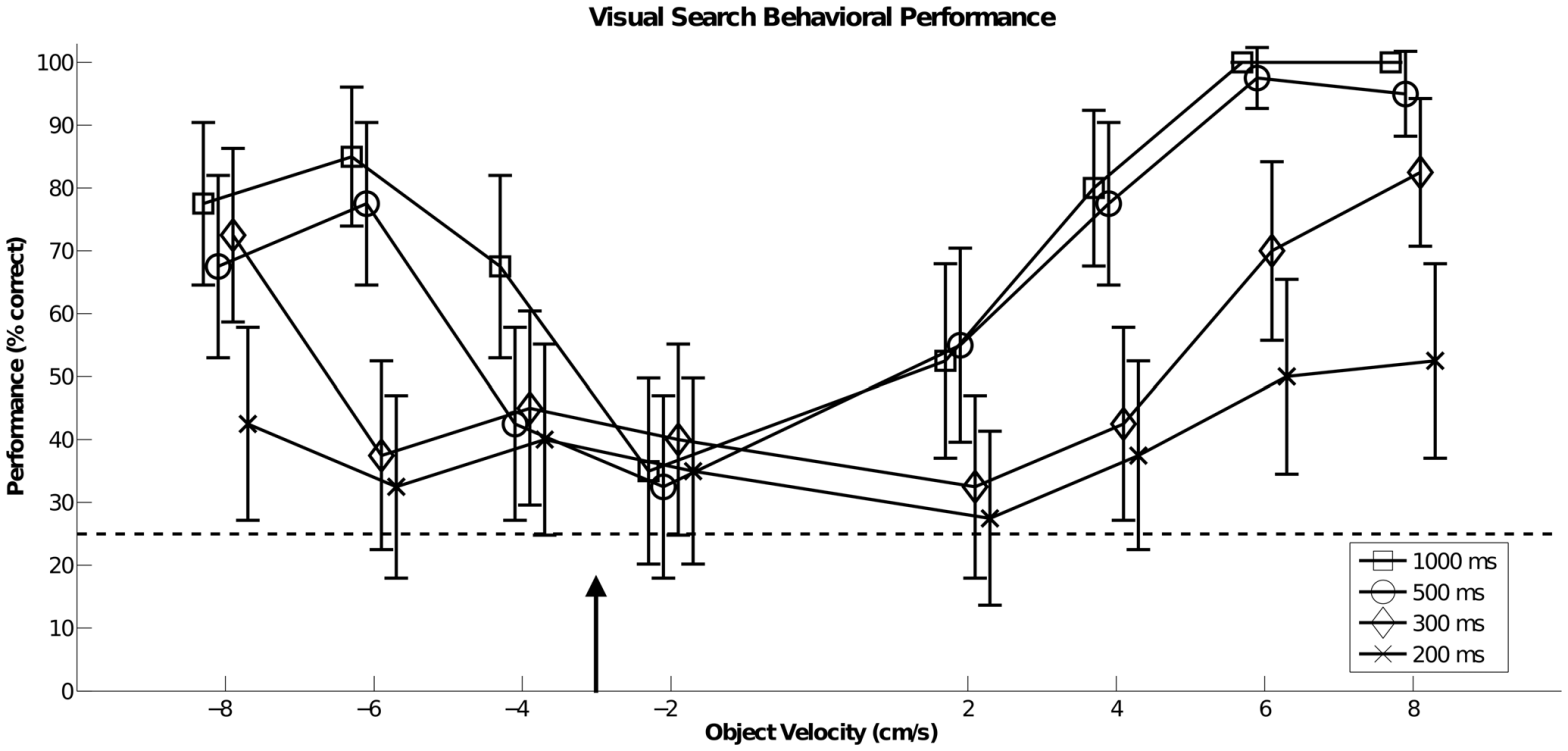
Visual Search (VS) behavioral performance as a function of absolute object velocity over varying stimulus motion durations (1000, 500, 300, 200) milliseconds. X-axis represents relative object velocity (cm/s) and y-axis represents performance (% correct). Error bars represent the 95 percentile confidence interval of the mean performance value. Dotted line at 25% indicates chance level. Arrow indicates observer motion velocity relative to objects (−3 cm/s). The difference between observer speed and object velocity is the relative object velocity shown in the display.

In the VS task, early in the stimulus motion period (starting at 19 ms) 10 Hz alpha power in the right hemisphere MT+ drops significantly below the alpha power in the Exp Only condition, suggesting an early recruitment of MT+ in this task. We hypothesize that this early decrease in power in MT+ may be explained by its involvement in the neural substrate of the flow parsing mechanism [25], by which the optic flow field due to self motion is subtracted from the retinal flow field as thus the independently moving object (the target) is detected. Our group has previously demonstrated that flow parsing could be the effective mechanism by which an observer in forward motion can detect an object moving in depth (as described in the VS task [24], [25]) Here we show that it does so in the first 300 ms of the stimulus in MT+ through observing 10-Hz alpha band power changes, and linking the changes to alpha inhibition. In Figure 8 we show that behavioral performance on the VS task was significantly degraded for stimulus duration shorter than 300 ms. There was no statistically significant difference in observers' performance for the stimulus duration of 1000 ms or 500 ms (two sample proportions test, p = 0.07). There was, however, a statistically significant difference when comparing overall performance between stimulus duration of 500 ms duration and a 300 ms (p<0.01) or 200 ms (p<0.01).

**Figure 9:**
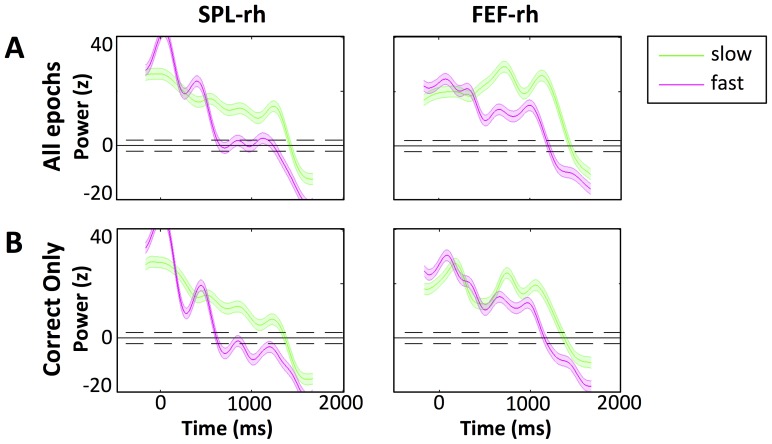
Time courses of 10 Hz alpha band oscillatory power in SPL and FEF with respect to the target speed. Fast (absolute speed greater than 5 cm/s, pink) and slow (absolute speed lower than 5 cm/s, green) speeds are shown in the VS task (A) averaged over all epochs and (B) averaged over correctly answered epochs. The y-axis represents the z-score of oscillatory power. The x-axis represents time in milliseconds relative to motion onset (0 ms). The blue line shows the 10 Hz power in the Exp Only condition with 2SD error shown in blue shade around the time course. The red line shows the 10 Hz power in the VS condition with 2SD error in red shade around the time course. The black horizontal line indicates z = 0, which is the average normalized baseline power. Dotted lines indicate z = 2 and z = −2 lines.

There was a large difference in performance for slow speeds of the target (absolute speed less than 5 cm/s) versus fast speeds (absolute speed greater than 5 cm/s) at all stimulus durations (Figure 8). We were interested to find if this performance difference manifests itself in the 10 Hz power in the fast and slow trials in the frontoparietal regions, which we suggest are involved in spatial attention in the VS task. After averaging over the 10 Hz power separately for fast and for slow target speeds, we found that the speed only affects the activation of frontoparietal regions SPL and FEF. In Figure 9A, the 10 Hz activation in SPL and FEF drops off earlier for the fast moving target (greater than 5 cm/s). We suggest that for slower target speeds, SPL and FEF perform additional validation checking 10 Hz of the candidate target sphere past 500 ms since slow targets are more difficult to detect, as shown through lower performance at slow speeds (Figure 8). Alternatively, it is possible that incorrect responses may have lead to the extended alpha power. However, we found little difference in activation when removing incorrect responses from the average (Figure 9B).

The inhibition hypothesis of alpha band power provided a way to measure, by rise and fall of the 10 Hz alpha power, the timings of inhibition of cortical processing of the VS and Exp Only Tasks. In both tasks the visual processing regions became engaged by 300 ms into the motion of the stimuli, but later (>300 ms) we see that alpha power diverged revealing significant differences in recruitment of these regions in task processing. Finally, we found different profiles of the 10 Hz alpha power in the frontoparietal regions, suggesting a different mode of processing among these ROIs. In the following section, we further explore the differences between the frontoparietal and visual processing regions by investigating the 10 Hz alpha synchrony.

### 2. Separable synchrony networks leading to separate function

In the previous section, we discussed how the inhibition hypothesis explains the 10 Hz alpha power in the visual processing regions and the differences in the activation between Exp Only and VS. The frontoparietal areas (MPFC, FEF, and SPL) followed a different pattern (Figure 5B). Here we discuss the differences in the phase-synchrony between these regions. Figure 7 illustrates two ROI clusters exhibiting 10 Hz alpha phase synchrony: one is defined by V3a-VIP-MT+ which is involved directly in motion processing and the other is defined by the frontoparietal ROIs, MPFC-FEF-SPL, involved in control and modulatory functions. We hypothesize that these two separate networks provide the underlying neural substrate for separate and independent mechanisms involved in solving the VS task. The visual-processing ROIs compute motion features related to the object while the frontoparietal ROIs compute which object to attend to. In this paper we focused on how these two networks interact at the 10 Hz alpha frequency. Results of the WPLI computation showed that the network of visual processing regions (V3a-MT+-VIP) and, separately, the network of the frontoparietal regions (SPL, FEF, MPFC), are synchronized in phase at 10 Hz throughout the stimulus-motion period (Figure 7). However, the individual areas between the two networks were not significantly synchronized. Several authors have proposed that activated cortical areas that intercommunicate during the task period may be synchronized in the 10 Hz alpha power through an oscillatory generator, such as the thalamus or other subcortical areas [2], [57]. Saalman and collaborators [58] have suggested that subcortical alpha rhythms underly the communication among cortical regions during task processing. It is possible that regions that show strong phase synchrony at 10 Hz alpha power may be jointly driven by the same “pacemaker” from the thalamus. This synchrony would preclude intercommunication during the stimulus motion among cortical areas within each of the two networks and indeed this lack of communication between component areas of the two networks is noted in the 10 Hz alpha synchrony in the VS task. The occipital-parietal alpha-band phase locking reported by Doesburg and colleagues [59] in an EEG study conflicts with our findings. However, their experiment cued the subjects to where the target object would be placed on the screen prior to the onset of the task. In the VS task, however, subjects are not cued to the location of the target. To detect the target, subjects must shift their attention during the motion-stimulus period from sphere to sphere without having prior knowledge of the probable object location. Thus we suggest that the occipital-parietal phase locking found in Doesburg and colleagues' study could be related to maintaining the stationary focus of attention.

### 3. Attention in the frontoparietal network

In the previous sections we discussed two independent networks of cortical regions, a visual processing network, involved in processing the motion stimulus, and a frontoparietal network, possibly involved in attention control. Our results showed that the regions in the frontoparietal network are asynchronous in the 10 Hz alpha-band with the regions in the visual processing network. In this section we address the potential roles of the regions in the frontoparietal network in the VS task. The prefrontal cortex (including MPFC) is associated with executive control function and working memory [60], [61]. Neurophysiological and human functional imaging studies suggest that the frontal eye field (FEF) [48], [54], [62] plays a decisive role in voluntary saccadic eye movements [54], and also in covert attention shifts [63], [64] required for target selection and/or attention control [65]–[67]. There is also strong evidence for the involvement of SPL in top-down spatial attention [68] and shift of attention [64], [69]–[71]. Taking into consideration the roles attributed to these ROIs in the frontoparietal network we propose a qualitative model of top-down attention control in the VS task: SPL is responsible in the switching of attention among objects (the moving spheres), and sends a signal to FEF to perform the actual attention shift, while MPFC is associated with in the attention buffer which provides the spatial working memory that records the candidate target locations that have been previously evaluated. MPFC is also involved in executive control and mediates top-down attention to the features (relative to target location) relevant to the task at hand. We found that when an object was located in the left visual hemifield, for example, the corresponding SPL and FEF regions in the opposite hemisphere (right hemisphere) had a decrease in 10 Hz alpha power. This indicates that SPL and FEF are both sensitive to aspects of the computations involved in searching for the spatial location of the target object (the independently moving sphere) while MPFC is responsible for the efficiency of this search (keeps track of the already visited locations).

## Conclusion

Using psychophysics and MEG we described in humans two cortical networks with different activation profiles of the 10 Hz alpha oscillations: a visual processing network containing the occipital and posterior parietal ROIs (V3a, MT+, VIP) and frontoparietal network (SPL, FEF, MPFC) computing spatial attention. Our data indicates that, although the 10 Hz oscillations are highly synchronous among regions within each network, there is no synchrony between regions in different networks. We suggest that this lack of synchrony could be driven by separate oscillations, supporting strong communication within these networks for visual processing processing and attention control.

Our results provide evidence for the hypothesis that the 10 Hz alpha oscillations indicate activation or deactivation of cortical regions. Early on in the visual processing regions (before 300 ms), alpha power is high, which suggests inhibition. Then, 10-Hz alpha power drops significantly after 300 ms, suggesting that regions become engaged in computing the VS task. In the frontoparietal regions, there is long and sustained 10 Hz alpha power during the stimulus motion period. We suggest that the alpha power in the frontoparietal network is used to modulate top-down attention.

We began to investigate how the results reported here on the VS task at the 10 Hz alpha oscillation relate to the profile of the gamma frequency bands, known to be implicated in processing sensory and cognitive tasks [72], and its cross-frequency coupling [73], [74] with theta and alpha bands. Because the faster gamma band frequencies allow for shorter transients, they are directly linked to the cortical computations [72], [75] and as such, these higher frequencies will provide further insight into the mechanisms in performing the VS motion task, and may shed light onto the cortical spatiotemporal orchestration of the higher level, cognitive, motion task.
